# Procedural justice and (in)equitable participation in climate negotiations

**DOI:** 10.14324/111.444/ucloe.3116

**Published:** 2025-02-06

**Authors:** Carola Klöck, Christian Baatz, Nils Wendler

**Affiliations:** 1 Center for International Studies, Sciences Po, Paris, France; 2 Department of Philosophy, Kiel University, Kiel, Germany

**Keywords:** climate negotiation, climate justice, delegation size, procedural justice, UNFCCC

## Abstract

Formally, state parties are equal in all United Nations negotiations. In theory, every state, regardless of its size, economic or political power, has the same opportunities and rights to participate. Nevertheless, United Nations negotiations, such as those on climate, are often considered highly unequal in practice. Many states struggle to meaningfully engage in complex and highly technical multilateral negotiations, including because their delegations are smaller. Here we examine delegation size in United Nations climate negotiations through a procedural justice lens. Starting from normative principles of procedural justice, we argue that equitable negotiations demand the capability of all parties to send a *sufficient* number of delegates – around 15. Using descriptive analysis of data on delegation sizes of recent Conferences of the Parties, we then highlight that many parties in practice send smaller delegations. Based on these results, we suggest two routes for making climate negotiations more equitable: (i) providing additional resources to poor states to increase their delegation size; and (ii) trimming the overall negotiation agenda to lower the sufficiency threshold.

## Introduction

Equity, fairness and justice are key concerns in United Nations (UN) climate negotiations. This includes procedural justice, usually understood as the ability of all affected stakeholders to participate in decision-making processes and influence the outcome [1–3]. In this context, the global climate change negotiations under the United Nations Framework on Climate Change (UNFCCC) are of prime importance. Although this process could a priori be considered equitable and fair, given its practice of consensus and its openness to all countries, the negotiations are by and large seen as fundamentally unjust in practice [2,4–6].^1^

This injustice is mainly related to the difficulties of smaller and poorer countries, such as the least developed countries (LDCs) or small island developing states (SIDS), to meaningfully engage in the climate change negotiations; they are ‘not equal partners in international negotiations on climate change’ ([7]: 264). The disadvantages for small states in multilateral negotiations are well documented in the negotiation and international relations literature, and are mainly related to small delegation size [5,8–10]. Admittedly, delegation size is a crude measure of negotiation capacity. Not all delegates are alike, and delegations may also comprise technical staff, security personnel or non-governmental organisation (NGO) members, who do not contribute to the delegation’s negotiation capacity [11,12]. Similarly, large delegations do not guarantee negotiation success [8,13]. Nevertheless, small delegations are disadvantaged compared to larger delegations, and very small delegations in particular struggle to engage meaningfully in complex multilateral negotiations such as those on climate change, as we discuss this in greater detail below.

Would larger delegations then automatically make for more just negotiations? Which principles of procedural justice should inform our assessment of international climate negotiations? We explore delegation size and its implications for procedural justice by combining empirical negotiation research and normative political philosophy – research strands that rarely intersect. This interdisciplinary lens allows us to confront philosophical criteria for procedural justice with empirical data on actual delegation size to assess the extent to which climate negotiations are procedurally unjust – and suggest ways to improve the negotiation process from a procedural justice perspective.

In the following section, we first outline the role of delegation size in negotiations regarding climate change and beyond, and then turn to principles of procedural justice, which suggest that parties should be able to send *sufficiently large* delegations. In the next section on delegation size at Conferences of the Parties (COPs) since 2015, we examine actual participation data for the last eight COPs (2015–2023); this analysis suggests that the climate negotiations do not meet the criteria for procedural justice outlined. In the final section on more procedurally just climate negotiations, we discuss these findings and suggest three ways to make climate negotiations more procedurally just: increasing the minimum delegation size; trimming the agenda; and potentially setting a limit on delegation size.

## Why delegation size matters

In UN negotiations the size of the delegation matters. According to Roberts and Parks ([5]: 16), the ‘importance of the number of attendees that developed and developing governments send to negotiations can […] not be overstated’. Larger delegations present several advantages in navigating ‘environmental mega-conferences’ such as the COP of the UNFCCC [14].

The climate summits are increasingly structured into multiple bodies and work streams to deal with the widening climate agenda. As a result, many meetings and consultations take place in parallel. During COP20 in 2014, one study observed ‘at least 17 meetings under five bodies […] taking place [simultaneously]’ – and this excludes closed negotiation meetings, or informal side events, press briefings or the like ([15]: 84). Clearly, more delegates can cover more meetings and more agenda items, while smaller delegations need to prioritise which meetings they attend [2,8,9,16,17].

At the same time, meetings often run late into the night, or even through the night. The final negotiation session of COP25, for example, overran by over 40 hours [6]. Larger delegations can better deal with such ‘negotiation by exhaustion’, [16], for example, by rotating the delegate(s) sitting in lengthy meetings that go over schedule [8,10,18,19].

The climate negotiations also increasingly use closed informal meetings, known as ‘informal informals’, contact or spin-off groups. These meetings tend to overlap with other – formal and informal – negotiation sessions and are scheduled haphazardly by chairs of the sessions [20,21]. It is much more difficult for smaller delegations to follow and engage in informal meetings and processes [20,22].

The sheer number of meetings and agenda items of any one COP also requires substantial technical and legal expertise to understand what is at stake, read through hundreds of pages of documents (such as text proposals or positions from other parties) and formulate one’s own positions [9,10,21]. Alongside all the documents produced and distributed in advance of negotiation sessions, there are also countless in-session documents ([21]: 25f). Small delegations simply do not have the time to read through all these documents ([5,23]). This ‘paradoxical information asymmetry’ has also been observed at the UN in general, where small states ‘are inundated with information they cannot process while simultaneously lacking access to crucial insider information’ ([24]: 11).

Furthermore, small delegations also do not have experts on every topic covered, as compared to larger delegations which typically have dedicated negotiators, or even teams of negotiators, for every major agenda item. In smaller delegations, in contrast, one negotiator covers several items [10,19]. Yet, substantial knowledge and understanding of the topic is a prerequisite for active participation and meaningful engagement, for making constructive proposals and contributing to discussions [10,25–27]. Accordingly, smaller delegations that lack such expertise ‘tend to get left in the dust as the discussions get more technical and go beyond the level of expertise of their negotiators’ ([21]: 24).

Finally, alongside the formal negotiations, the COPs also boast an impressive array of parallel events ‘on the side’. Side events, pavilions, exhibitions or press briefings are used to inform the public on progress in the negotiations, to network, to build capacity and to understand the positions of other parties [9,18,28]. Attending such side events again requires human resources, not least because often, these informal spaces are physically removed from the formal negotiation spaces ([17]: 221f).

The disadvantages that small delegations face seem unjust. The following section outlines an account of procedural justice that allows specifying in what way differences in delegation sizes are unjust – and in what way they are not.

## Procedural justice in the climate negotiations

Procedural justice aims at designing fair procedures based on normative principles (e.g., [3]). Scholars have investigated the fairness of various procedures related to climate change, for example, on the level of national and local implementation [29] or in the context of international adaptation funding [30]. As the most relevant stage for multilateral decision-making in international climate policy, the UN climate negotiations have also been analysed from a procedural justice perspective [2,31,32]. In general, one can think of two reasons why procedural justice is relevant. The first reason is that procedural justice is inherently valuable because parties have a moral right that the procedures conform to certain normative standards. The second reason is instrumental: fair procedures are not just valuable in themselves. They can also help increase the likelihood the effectiveness of procedures, for example, because they help resolve the problem of ‘reasonable disagreement’ in climate negotiations ([33]: 787; see also [2]).

In the following, we build on Tomlinson’s account of procedural justice in the UNFCCC negotiations [2] because it is comprehensive and detailed, both in the theoretical foundations of his normative approach and its practical applications, with many examples of what procedural justice may demand in climate negotiations. His account of procedural justice is based on the idea of political equality. According to Tomlinson, political equality among parties in climate negotiations is based on two principles: all parties should have (i) equal status and (ii) equal opportunity to influence decisions. Let us look at these principles in turn.

First, equal status is about the basic recognition and respect of all parties as equals. Formally, state parties are equal in all UN negotiations. Decisions are taken by consensus. At least in theory, every party, regardless of its size, economic or political power, can table proposals, engage in discussions, and potentially block decision making. However, for Tomlinson, equal status goes beyond formal equality. It is also about how parties are treated in the negotiations and whether all delegations are met with the same respect ([2]: 116–17). Second, Tomlinson argues that parties should not just have equal status but also equal opportunity to influence decisions, ‘where influence concerns the amount of control that a decision-maker has over the outcome of the decision’ ([2]: 120).

We consider equal status and equal opportunity to influence decisions to be two high-level principles that are capable of capturing the more fine-grained criteria of procedural justice commonly discussed in the literature. For example, under equal status, Tomlinson discusses procedural issues related to whether parties are treated respectfully ([2]: 116), while other scholars have a distinct criterion for respect (e.g., [32]: 788). The same holds true for other criteria like information or transparency, which can be derived from and discussed under Tomlinson’s high-level principles.

In general, it must be noted that many approaches to procedural justice tend to converge at the level of the substantive concepts they use to assess procedural justice. While scholars differ in what they treat as distinct criteria or how they interpret them, many approaches share some basic ideas of what procedural justice demands. For example, one can usually find criteria related to the idea of transparency or correctability [32,33]. The appropriateness of the approach one chooses also depends on what one seeks to analyse. For the purpose of this paper, we mainly have what one may call an agent-centred view: what matters to us is what a given delegation needs in order to participate in the negotiations. To be clear, what a delegation needs to achieve this depends heavily on how the procedures themselves are designed, such as how transparent they are or how information is provided. Hence, the different criteria of procedural justice are interdependent. To give a simple example: the less transparent procedures are, the more resources a delegation needs to keep pace with the negotiations. Thus, one way to tackle procedural justice issues related to delegation size is to make the negotiations more accessible (see Toward more procedurally just climate negotiations).

For now, however, we leave the design of procedures aside. Instead, we want to approach the problem from the delegations’ side. For Tomlinson, the parties’ opportunities to influence decisions depend on their resources and capabilities [2]. Resources in this context can refer to many things: monetary resources, knowledge, social capital, experience, personnel – everything that is required to follow and engage in complex and lengthy negotiations.^2^

Capabilities refer to ‘the various capacities or abilities that actors have to perform a certain function, where a function is an activity that an agent can undertake’ ([2]: 120). While it seems clear that resources like money or knowledge are of great importance for parties to participate, Tomlinson argues that what matters is not the resources themselves but rather parties’ capabilities to reach certain functions, such as ‘forming opinions, making judgements, and advocating interests and positions’ ([2]: 123). Resources, seen this way, are just one determinant of what agents are de facto able to do. The critical point is that they have ‘sufficient resources to [be able to] participate on equal terms’ ([2]: 125).

The number of party delegates is a central – if not the only – resource for reaching those functions. Following the evidence on delegation size summarised in the section on why delegation size matters, a certain number of delegates seems to be a necessary condition for having equal opportunities to influence decisions. With just a few delegates a party will have, for example, fewer options to advance its specific position in multiple negotiation streams and in public compared to a medium or large delegation.

One may criticise Tomlinson’s demand for *equal* opportunity to influence decisions. The size of countries within the UN varies massively. Do we really think that it would be fair for Brazil, Nigeria and Malta to have the *same* opportunity to influence decisions even though the latter represents much fewer people? A less ambitious principle that we propose demands *sufficient* opportunity to influence decisions. Whatever may speak for or against these principles in ideal theory, we think that calling for *sufficient* opportunity to influence decisions better reflects political realities, while still being an improvement compared to the status quo. Equalising these opportunities will be more difficult to establish compared to enabling all parties to participate properly despite remaining differences in opportunities. Tomlinson’s framework does offer ideas to qualify what participating *properly* could mean under a principle of sufficient opportunity to influence decisions. For example, sufficient opportunity could mean that parties are not required to be able to influence *all* decisions they are affected by. Instead, they should be able to influence at least those decisions that may affect their autonomy ([2]: 95; [34]: 4).^3^ But even on this more modest principle, parties will need sufficient resources to not just attend the negotiations but to have some kind of impact on the decisions. Regardless of how one further operationalises this, it seems plausible to assume that parties will need to be able to send a sufficient number of delegates to have any impact at all.^4^

What would be a *sufficiently* large delegation? Assuming that each delegation should be able to (i) attend all relevant formal and informal meetings with at least one person, (ii) attend some side events, (iii) engage with civil society and the media, and (iv) get some rest, we believe that a minimum number of around 15 delegates is required at present. Why 15 delegates? Current evidence suggests that a delegation with less members will struggle to fully engage in current negotiations. To be clear, any definition of a sufficiency threshold is somewhat arbitrary, and there is no straightforward method to determine at which number a delegation is sufficiently big. That being said, we base our threshold of 15 delegates on previous research on delegations and negotiation capacity. In a survey of Alliance of Small Island States (AOSIS) negotiators, a majority of respondents indicated their country should send four to five delegates (41% of respondents) or six to 10 delegates (34.5%) [35]. Even four or five delegates could not attend all meetings that take place in parallel, as mentioned earlier. Accordingly, Falzon ([10]: 8) cites an LDC negotiator who suggests 10 delegates is the minimum. Even a 10-person-delegation may be too small; the authors of the UNFairplay report have ‘observed parties of around 17 delegates being seriously stretched and unable to participate fully in negotiations’ ([23]: 14). Given these insights from the literature, we chose 15 delegates as a threshold below which it becomes very likely that the respective delegation will struggle in the negotiations. As the real world with its complexities does not exhibit a clear-cut threshold for sufficient delegation size, this is to be seen as a rough estimate, and 15 delegates seems to be at the lower end of a sufficiency spectrum.

Finally, note that Tomlison’s principles of equality of status and of opportunity start from the current basic UNFCCC structure of consensus decision-making among formally equal parties. Rather than take the institutional framework for granted, we could also criticise the UN system (including the UNFCCC process) as such [36–38]. We do not want to enter this global justice debate, but instead simply recall that climate negotiations take place against the background of a very unjust situation: while climate change is mostly caused by the emissions of wealthy and powerful states, poor people – who hardly contribute to nor benefit from greenhouse gas emissions – are most affected by its impacts [39,40]. We should therefore pay specific attention to which parties (and indirectly, the people they represent) send small delegations. We therefore now turn to analysing actual delegation size at recent COPs.

## Delegation size at COPs since 2015

To understand delegation size in practice, we use the official lists of participants, focusing on the last eight COPs since 2015. This time period gives a current overview and takes into account the overall increase in delegation size since Paris [41].

We do note that these delegation sizes are only a *proxy* of negotiation capacity. Numbers may be misleading; not all delegates listed in the official lists of participants are technical negotiators. They could also be security or logistics staff, or civil society members accredited through the government, but not contributing to the negotiations [11,12]. Some government delegations also include representatives from the private sector, including fossil fuel companies and other carbon-intensive industries [42,43]. Nevertheless, we maintain that delegation size is a widely used and useful proxy, and that smaller delegations are disadvantaged, as explained above.

Delegation size varies significantly, both across countries and over time. Overall participation numbers increased significantly since the first COP in 1995. Delegations comprised on average six to seven delegates in the first years of the negotiations (1995–1999). This grew to nearly 24 delegates in the period after the entry into force of the Kyoto Protocol (2005), to 61 delegates in the 9 years since Paris (2015–2023).

At the same time, for most countries, delegation size varies significantly from one year to the next. Let’s look at Rwanda, a country whose average delegation size (60) is very close to the overall average. While the Rwandan delegation indeed comprise 60 delegates on average for the recent climate summits, it ranged from only 11 delegates at COP23 (Bonn, Germany) to 191 delegates at COP28 (Dubai, United Arab Emirates). For other countries, the year-to-year variation is even more striking. The largest ranges in delegation size (of 1000 and more) are found for Morocco and United Arab Emirates, which can be explained by their extremely large delegations when these countries hosted a COP. The Indian delegation displays a similarly large variation, ranging from 35 at COP24 and COP25 to 808 at COP28.

**Figure 1:**
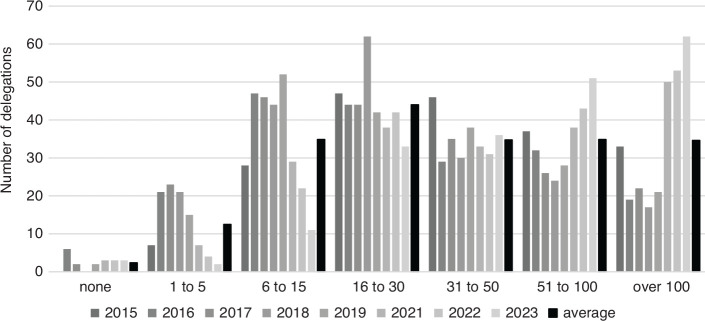
Frequencies of delegation size for COP21 (2015) to COP28 (2023). Based on lists of participants.

These examples already indicate the large variation of delegation size across countries. Figure 1 shows the frequencies of delegation sizes for the past eight COPs. Across all COPs, 31 countries have average delegations below the ‘sufficiency threshold’ of 15 discussed earlier. If we look at individual COPs, the number of ‘insufficiently large’ is significantly larger: around 70 countries had delegations of 15 or less at COPs 22 through 25. At COP21, and since COP23, the number of such small delegations was, however, lower, at around 35, and has decreased to only 16 at the most recent COP28 (Dubai, 2023). At the other extreme, we also have many extremely large delegations: 35 delegations per COP are larger than 100 delegates – and on average four delegations per COP comprise even 300 or more delegates. Indeed, there seems to be a trend toward such ‘mega-delegations’: while three countries had sent more than 300 delegates to COP21 in Paris (2015), the number of such delegations increased to 10 at the most recent COP.

Who are the countries sending delegations that seem insufficiently large? In fact, a large number of countries are concerned, many of which are very small and/or relatively poor – but even larger and richer countries are under-represented on at least some occasions. In total, 12 countries were absent for at least one COP: San Marino (four times), Afghanistan, Myanmar (three times each); Bolivia, Eswatini, Kiribati, Moldova, Niue, North Macedonia, Syria, Saint Vincent and the Grenadines, as well as Trinidad and Tobago (once each). When we consider delegations of 15 or less, the number of countries concerned increases to 105 – more than 50% of all parties are thus under-represented at least occasionally. (For a full list, see Table A1.)

**Figure 2:**
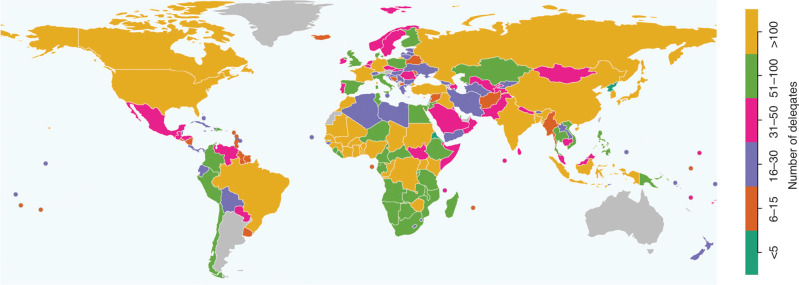
Average delegation size for COP21 to COP28. Based on lists of participants.

Because delegation size is so variable, we may instead consider average delegation size (see Fig. 2). Here, 31 parties have delegations that are on average below 15. The smallest average delegations come from very small countries: on average, San Marino and North Korea sent only three delegates; Eritrea, four delegates; and Liechtenstein, five delegates. Other examples of countries with small average delegations include, for example Guyana, Nicaragua, Mauritius, Nauru and Iceland. Yet, we also have some very small countries that manage to send more delegates Tuvalu, with a population of only 11,000, sent 26 delegates on average. Palau (population of 18,000) sent 29 delegates on average. The average delegations of Nauru (population of 13,000) and the Cook Islands (population of 15,000) are fairly close to our sufficiency threshold, with 14 and 13 delegates, respectively. Similarly, some of the countries with the largest average delegations are relatively poor: when we exclude Morocco and the United Arab Emirates, who had extremely large delegations when they served as COP presidents, Brazil had the largest delegation on average (upper-middle-income, 352 delegates). It is followed by the Democratic Republic of the Congo (low-income country, average delegation of 298) and Côte d’Ivoire (lower-middle-income, average delegation of 282).

**Figure 3:**
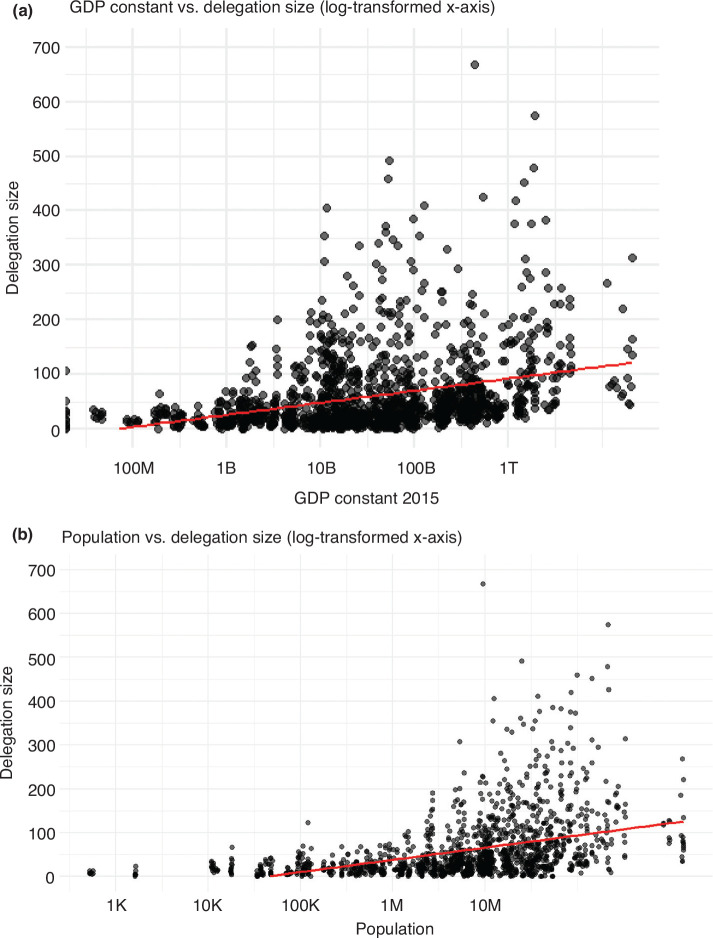
Average delegation size by income (a) and by population size (b). Based on lists of participants.

To establish more robustly whether there is a link between delegation size and income and population size, Fig. 3a plots average delegation size by income. We do the same in Fig. 3b with population size. Note that both income and population are log-transformed.

In line with previous research that suggests a strong link between income and delegation size [8,9], including for multilateral negotiations beyond climate change [44,45], we also find that richer countries tend to send larger delegations (Spearman’s rank correlation coefficient of 0.38). However, we do see a lot of variation in our data, and many very large delegations are in fact from low- or lower-middle-income countries, as mentioned earlier.

We also find a lot of variation for population (Fig. 3b), especially for larger countries. On the other end of the spectrum, we see clearly that the smallest countries send the smallest delegations on average. Accordingly, the correlation between delegation size and population size is stronger than for income (Spearman’s rank correlation coefficient of 0.52) and has become stronger over time (not shown).

To some extent, under-representation at the individual country level is mitigated by coalitions. Indeed, as for any multilateral negotiations, countries do not typically negotiate as individual countries, but through negotiation groups [46,47]. Coalitions help increase negotiation capacity and bargaining power and are therefore particularly relevant for countries with smaller delegations ([25]: 48). However, coalitions represent compromise positions, which may be relatively far away from the preferences of individual coalition members. In addition, agreeing on those compromise positions again requires negotiation and coordination, and therefore resources and capacity, which vary between coalition members. The same inequalities that characterise the overall climate negotiations are also found *within* coalitions [47,48].

The above analysis serves mainly illustrative purposes; our aim here is not a comprehensive and systematic analysis of delegation size and its drivers. The analysis does, however, indicate some tendencies: over time, delegations have increased in size, with most countries sending dozens of delegates to COPs. Nevertheless, some countries are present with only very few delegates – or may even be completely absent. Although we find that smaller countries also send smaller delegations on average – as is to be expected – we find many exceptions, and overall strong year-to-year variation. While our analysis thus does not suggest that a certain category of countries (such as small or poor countries) are *systematically* under-represented at COPs, we do find that a significant minority of countries send only a few delegates, and that over 50% of all countries are under-represented at least at individual COPs. From our analysis, we cannot draw any firm conclusion on why countries send only very few delegates. To some extent, delegation size also reflects political salience and domestic circumstance [18]. Nevertheless, given the number of countries concerned, we assume that some countries want to, but cannot, send more delegates [49]. These parties are unjustly disadvantaged in COPs because of their small delegations.

## Toward more procedurally just climate negotiations

Delegation size clearly matters; larger delegations have more opportunities to participate in (climate) negotiations and influence their outcomes. While delegation size varies significantly across countries and over time, we find that a significant number of parties have insufficient negotiation capacity for at least some climate COPs. In particular, smaller countries (in terms of population size) tend to send smaller delegations. Assuming that coalitions only partially compensate for insufficient delegates and that many parties which participate with only few delegates in COPs lack the capacity to send more, negotiations are unjust in this regard.

In order to make the UNFCCC negotiations more procedurally just, we discuss three suggestions. First, providing additional resources to poor parties will increase their delegation size and hence negotiation capacity. Second, trimming the negotiation agenda will lower the sufficiency threshold, that is, it will allow parties to effectively negotiate with fewer delegates than at present. A trimmed agenda might also make it easier to limit delegation size for all parties. The following briefly elaborates on each measure.

The UN already supports poorer parties. It established a Trust Fund for Participation in the UNFCCC Process to enable developing countries, in particular LDCs and SIDS, ‘participate fully and effectively in the climate change negotiating process’ [50]. The Trust Fund finances the participation of two delegates from eligible countries (with a per capita income under a given threshold) for COPs, and three delegates for LDCs and SIDS. Many parties would not be able to attend negotiations, in particular subsidiary meetings, at all without this support [10]. But to really enable all parties ‘to participate fully and effectively’, the fund would need to at least quadruple its support,^5^ provided that a minimum delegation size of around 15 adequately reflects negotiation reality. Such a massive increase in funding is very unlikely. In addition, it does not help small but (relatively) rich countries, which are the most under-represented, as discussed earlier. We therefore propose coupling increased support regarding poor parties’ negotiation capacity with simplifying the negotiation process.

The sheer size and complexity of the climate ‘mega-conferences’ have been criticised repeatedly [41]. While such mega-conferences can galvanise media, public and political attention and gather momentum, it is questionable to what extent this leads to concrete action – that is, whether they are worth ‘the effort, money, and carbon footprint’ [6,51]. There are thus calls on the UNFCCC to rethink its negotiation structure, ‘which, in its formal work and agendas, has become unwieldy and routinised, heavy in its carbon footprint, and out of step with the scale of urgency’ ([52]: 601; see also e.g., [41]).

Already in 2013, observers noted that there is significant room for improving the efficiency of the negotiation process, and suggested the UNFCCC could ‘streamline its work programme, cut sessions, eliminate overlaps, and delete agenda items’ ([53]: 251). Although such a reform would be politically difficult, fewer sessions and a reduced agenda could increase the chances of even small delegations to participate effectively in negotiations. In addition, a trimmed agenda reduces complexity and increases transparency because it makes it easier for parties, observers and the media to keep track of various meetings and negotiation streams. And smaller delegations mean fewer greenhouse gas emissions.

Parts of the negotiations, especially those under more technical meetings and constituted bodies, could also be ‘outsourced’ to virtual meetings that take place outside of COPs. As a result of the COVID-19 pandemic, many meetings, including some negotiation sessions, have been turned into a virtual format. Online meetings also present significant challenges, notably for smaller and poorer countries [54–56]. But online meetings also create new opportunities: they allow wider participation by reducing participation costs – in terms of both, financial and time investment [54,55]. While certainly not a replacement for COPs, the virtual format seems appropriate for some meetings and would help with trimming the workload of the annual COPs. To ensure proper participation of poorer and smaller countries in such online meetings, appropriate technical support, such as providing meeting spaces with good Internet connections, is indispensable. Similarly, the high-level segment and ministerial involvement could also be shifted to other settings, such as ‘Global Climate Action Weeks’, as proposed by Müller et al. [41].

A further, more radical, measure to reduce the complexity (and resource-intensity) of COPs is to limit delegation size and set a maximum number of delegates parties can send. Müller et al. [41] suggest that COPs with a total participation of around 5000 technical negotiators seem more manageable and productive than the current mega-events of up to 100,000 participants. A maximum delegation size would also mean fewer formal or informal meetings, thus contributing to reducing and then stabilising the minimum number of delegates required. However, setting a maximum number of delegates is probably not politically feasible at the moment. As political feasibility is a dynamic phenomenon [57], this calls for lobbying decision-makers to seriously consider such a measure. Limiting the agenda and simplifying the negotiation process may facilitate these efforts.

## Conclusion

Procedural justice requires even countries with very small populations to be able to meaningfully participate in decision-making, particularly when these countries – such as SIDS or LDCs – are particularly affected by the outcomes of that decision-making. In practice, parties that can send only small delegations are disadvantaged in multilateral (climate) negotiations. We showed that a substantial number of parties may indeed be unable to fully engage, assuming that delegations of around 15 negotiators seem necessary at present to follow all negotiation streams, as well as engage in side events and with the media.

We discuss three measures to improve climate negotiations from a procedural justice perspective: first, providing more financial support to allow all parties to send more than just two or three delegates. Second, trimming the climate agenda, and outsourcing some negotiation streams to (virtual) meetings. Finally, a limit on delegation size would reduce the complexity and resource intensity of the process. These two latter measures would allow even smaller delegations to fully engage in COPs, thus reducing the ‘sufficiency threshold’.

Delegation size is only one – if central – aspect of procedural justice in climate negotiations. Clearly, there are other factors that also influence how engaged different parties are and to what extent they are able to shape the negotiation process [13]. For example, even a small number of delegates can achieve a lot when they are experienced and knowledgeable. Small delegation size may also reflect lack of political will and interest in the negotiation process, rather than a lack of resources [18,58]. Finally, we want to emphasise that a procedural justice perspective allows for asking broader questions that we have not attempted to address in this paper. Most importantly, even if all parties were represented by sufficiently, or even equally, large delegations, further research should analyse whether the process is unfair in that it is a process between states. A starting point for such a broader analysis would be to ask whether the negotiations adequately represent all citizens and stakeholders, or whether indigenous peoples, future generations or non-humans are marginalised and un(der)-represented in intra-state climate negotiations. From this perspective, true procedural justice might thus require a reform of the UNFCCC.

## Data Availability

All data generated or analysed during this study are included in this published article (and its supplementary information files). See Table A1.

## Notes

1 For the purposes of this paper, we use the terms equity, fairness and justice synonymously to refer to morally objectionable procedural aspects of the climate negotiations.

2 Note that these resources are what Tomlinson calls internal resources, and he acknowledges that external resources, such as a state’s economic or geopolitical power, also matter in how parties can influence negotiations ([2]: 124). With this distinction in mind, our analysis focuses on internal resources. Arguably, although clearly influenced by the external resources of a party, it is even harder to change the distribution of external resources than it is to provide parties with more internal resources.

3 This again would need to be qualified. We thank Kilian de Ridder for drawing our attention to this point.

4 In the context of this paper, we do not engage further with the question of what exactly it means to be able to have an impact on decisions. Still, we want to emphasise that being able to impact decisions will often be a stronger demand than, for example, simply being able to voice one’s opinion. We can imagine that a very small delegation gets the opportunity to voice their concerns, yet this remains solely symbolic if they cannot properly follow the negotiations, formulate their positions or intervene in crucial moments.

5 Quadrupling support would enable SIDS and LDCs to send at least 12 delegates paid through the Trust Fund, still below but close to the minimum delegation size of 15.

## Appendix

**Table A1.** Delegation size for COPs 21–28. Based on lists of participants

Country	COP21	COP22	COP23	COP24	COP25	COP26	COP27	COP28	Average
Afghanistan	26	5	11	16	22	0	0	0	10
Albania	21	3	4	14	5	15	14	33	14
Algeria	44	20	24	22	36	24	65	2	30
Andorra	21	4	5	5	8	8	9	11	9
Angola	71	31	6	26	22	102	96	188	68
Antigua and Barbuda	9	9	10	5	15	31	23	28	16
Argentina	23	24	23	11	65	121	44	23	42
Armenia	27	4	13	7	12	47	51	52	27
Australia	45	45	34	30	20	94	82	105	57
Austria	43	39	37	50	36	37	44	48	42
Azerbaijan	66	5	9	7	7	83	13	213	50
Bahamas	24	6	2	4	10	14	57	29	18
Bahrain	31	19	7	12	9	62	102	90	42
Bangladesh	44	95	35	83	143	295	68	110	109
Barbados	10	2	4	8	6	16	28	68	18
Belarus	11	8	2	7	7	10	8	47	13
Belgium	74	39	33	29	42	57	56	55	48
Belize	18	15	22	17	28	33	32	33	25
Benin	137	92	130	139	116	108	166	126	127
Bhutan	17	10	10	16	32	31	15	28	20
Bolivia	26	4	8	20	0	27	38	32	19
Bosnia and Herzegovina	12	1	10	14	6	24	16	31	14
Botswana	39	36	31	53	27	52	108	117	58
Brazil	217	100	128	107	172	479	574	1037	352
Brunei	10	5	7	18	3	14	14	68	17
Bulgaria	25	7	5	26	13	13	23	79	24
Burkina Faso	204	163	162	90	104	109	179	159	146
Burundi	49	21	34	12	32	38	117	129	54
Cabo Verde	25	24	17	16	37	21	34	23	25
Cambodia	40	21	25	33	26	61	88	99	49
Cameroon	167	67	114	39	50	52	77	114	85
Canada	287	202	161	126	145	276	377	176	219
Central African Republic	50	38	51	36	27	53	107	88	56
Chad	86	51	52	57	62	201	210	118	105
Chile	149	66	26	31	136	63	69	39	72
China	268	78	82	90	76	60	65	221	118
Colombia	78	19	13	17	26	149	98	67	58
Comoros	70	44	21	12	10	50	65	70	43
Congo	175	126	308	164	165	170	237	145	186
Cook Islands	14	12	12	12	11	10	16	18	13
Costa Rica	45	24	19	15	52	43	22	23	30
Croatia	25	12	10	10	26	17	24	22	18
Cuba	27	7	6	8	8	18	14	92	23
Cyprus	16	6	5	4	5	13	27	29	13
Czechia	49	17	16	26	46	55	75	50	42
Côte d’Ivoire	215	361	492	208	348	169	226	234	282
Democratic Republic of Congo	304	161	340	237	293	373	459	213	298
Denmark	102	41	32	50	43	116	96	130	76
Djibouti	62	42	16	16	14	17	55	62	36
Dominica	5	8	7	5	4	8	11	20	9
Dominican Republic	88	29	85	35	100	69	76	56	67
Ecuador	46	16	15	8	45	54	32	16	29
Egypt	84	29	35	28	31	115	155	142	77
El Salvador	29	15	10	14	9	10	16	8	14
Equatorial Guinea	95	91	78	24	58	28	35	68	60
Eritrea	3	4	1	3	3	3	7	7	4
Estonia	22	24	25	24	18	21	24	27	23
Eswatini	22	0	16	14	14	38	16	29	19
Ethiopia	64	48	33	42	48	72	131	221	82
European Union	128	89	76	83	125	122	118	120	108
Fiji	43	35	74	60	35	47	30	64	49
Finland	74	36	38	51	57	49	61	54	53
France	383	287	177	188	124	197	186	259	225
Gabon	32	35	22	21	13	125	99	108	57
Gambia	40	53	69	93	69	151	153	98	91
Georgia	43	7	10	12	26	44	36	55	29
Germany	117	105	230	153	102	120	118	259	151
Ghana	126	69	159	111	106	337	155	91	144
Greece	26	8	7	8	38	32	44	62	28
Grenada	11	11	7	6	11	19	26	31	15
Guatemala	55	38	59	25	49	50	32	43	44
Guinea	99	173	355	406	159	109	85	133	190
Guinea-Bissau	25	12	29	14	21	25	46	46	27
Guyana	11	9	4	4	5	12	8	20	9
Haiti	15	26	18	12	26	46	30	24	25
Honduras	70	16	31	54	83	74	13	48	49
Hungary	36	16	24	22	27	30	21	53	29
Iceland	17	6	7	8	7	27	19	20	14
India	185	94	45	35	35	134	70	808	176
Indonesia	187	124	158	191	163	156	158	206	168
Iran	17	16	30	15	15	25	25	21	21
Iraq	68	44	56	51	46	122	235	252	109
Ireland	49	21	24	33	20	86	66	75	47
Israel	77	27	18	13	20	229	227	92	88
Italy	78	73	55	49	39	66	81	127	71
Jamaica	12	13	12	13	13	28	12	24	16
Japan	167	103	109	116	138	225	152	238	156
Jordan	26	14	17	15	9	74	134	188	60
Kazakhstan	40	10	13	27	19	192	78	330	89
Kenya	96	107	62	72	95	308	386	292	177
Kiribati	28	13	33	20	13	0	29	64	25
Kuwait	36	85	25	29	29	64	40	55	45
Kyrgyzstan	61	3	13	27	19	58	9	48	30
Laos	20	7	14	18	9	12	21	39	18
Latvia	26	9	5	20	19	26	26	42	22
Lebanon	43	8	3	5	9	12	19	34	17
Lesotho	27	33	19	17	28	44	19	41	29
Liberia	45	45	30	28	51	147	201	100	81
Libya	5	14	11	3	2	26	32	63	20
Liechtenstein	6	5	5	4	4	9	3	7	5
Lithuania	30	7	12	27	8	31	22	33	21
Luxembourg	49	11	20	25	24	27	20	21	25
Madagascar	91	60	70	86	20	76	106	86	74
Malawi	40	22	39	23	54	138	141	154	76
Malaysia	37	27	33	16	20	27	74	157	49
Maldives	24	21	26	24	13	56	58	42	33
Mali	130	178	102	91	66	118	101	54	105
Malta	17	14	10	8	6	21	24	34	17
Marshall Islands	36	26	41	28	28	35	29	39	33
Mauritania	115	88	104	37	59	72	172	198	106
Mauritius	13	6	4	10	8	14	7	12	9
Mexico	27	50	60	33	35	25	29	32	36
Micronesia	22	15	28	18	15	5	30	22	19
Moldova	17	0	3	11	5	9	18	10	9
Monaco	27	21	12	20	16	23	17	16	19
Mongolia	31	2	14	20	11	46	56	68	31
Montenegro	25	9	11	17	17	24	25	21	19
Morocco	355	1592	253	104	137	75	205	411	392
Mozambique	64	18	32	29	48	75	139	162	71
Myanmar	27	17	17	16	20	0	0	0	12
Namibia	93	17	31	50	37	84	138	98	69
Nauru	13	10	17	14	18	12	15	14	14
Nepal	29	29	26	39	14	75	76	106	49
Netherlands	36	22	24	40	40	37	48	40	36
New Zealand	36	25	20	21	19	16	19	26	23
Nicaragua	10	6	7	4	7	5	9	6	7
Niger	142	127	54	55	77	117	169	39	98
Nigeria	86	81	77	141	65	117	169	426	145
Niue	4	0	5	3	6	4	13	24	7
North Korea	8	2	2	3	3	3	4	2	3
North Macedonia	22	0	2	10	7	16	9	20	11
Norway	69	51	33	36	41	47	40	42	45
Oman	26	19	10	16	15	16	82	160	43
Pakistan	73	32	42	17	16	10	85	74	44
Palau	41	21	29	4	7	27	37	67	29
Palestine	38	18	6	11	13	25	31	17	20
Panama	54	23	10	15	26	47	37	27	30
Papua New Guinea	56	15	28	19	35	110	105	100	59
Paraguay	57	32	26	30	45	30	29	61	39
Peru	252	67	50	29	67	50	64	36	77
Philippines	138	51	65	28	8	23	29	247	74
Poland	45	46	77	211	38	37	65	62	73
Portugal	36	54	23	31	51	44	53	90	48
Qatar	87	64	28	39	39	174	110	190	91
Romania	21	12	19	25	16	49	62	75	35
Russia	260	55	71	54	52	312	150	452	176
Rwanda	20	34	11	19	18	61	129	191	60
Saint Kitts and Nevis	8	7	4	4	9	11	17	42	13
Saint Lucia	19	15	27	14	14	15	21	33	20
Saint Vincent and the Grenadines	10	10	9	7	6	3	0	11	7
Samoa	10	10	11	17	17	14	31	49	20
San Marino	6	0	0	3	0	13	3	0	3
Sao Tome and Principe	18	20	11	4	3	28	9	27	15
Saudi Arabia	35	36	42	38	34	60	44	49	42
Senegal	211	281	155	171	108	99	245	336	201
Serbia	12	7	15	23	20	43	35	34	24
Seychelles	44	42	56	47	34	53	40	123	55
Sierra Leone	38	39	60	32	44	113	100	117	68
Singapore	32	29	31	30	31	51	60	60	41
Slovakia	33	36	23	38	42	40	36	40	36
Slovenia	29	6	3	25	16	49	26	29	23
Solomon Islands	22	21	35	22	15	14	38	53	28
Somalia	10	2	3	7	8	22	97	195	43
South Africa	143	69	76	50	47	56	81	131	82
South Korea	204	100	79	80	81	137	114	113	114
South Sudan	15	8	27	19	15	34	51	107	35
Spain	46	59	36	46	172	84	106	75	78
Sri Lanka	45	17	10	14	12	52	38	66	32
Sudan	76	173	109	172	121	236	130	23	130
Suriname	11	3	14	15	9	22	16	29	15
Sweden	60	50	49	42	38	48	46	56	49
Switzerland	31	25	25	17	23	21	23	26	24
Syria	2	0	11	3	2	4	10	31	8
Tajikistan	15	4	10	10	5	56	77	115	37
Tanzania	17	29	35	31	33	127	193	209	84
Thailand	89	85	63	73	75	109	61	92	81
Timor-Leste	15	17	17	21	15	30	37	49	25
Togo	112	91	96	92	60	128	215	144	117
Tonga	16	15	19	26	42	11	53	69	31
Trinidad and Tobago	3	3	0	4	5	16	12	10	7
Tunisia	49	136	49	40	82	112	135	85	86
Turkey	125	154	86	81	81	376	163	420	186
Turkmenistan	7	3	1	5	4	11	5	95	16
Tuvalu	35	23	32	21	18	24	26	31	26
Uganda	93	100	152	61	121	219	241	275	158
Ukraine	39	19	19	20	17	55	23	55	31
United Arab Emirates	127	189	104	73	93	176	1073	668	313
United Kingdom	93	46	45	52	48	230	93	72	85
Uruguay	17	10	10	8	14	15	21	22	15
USA	147	93	48	44	78	165	136	314	128
Uzbekistan	4	2	1	5	6	40	43	267	46
Vanuatu	31	19	39	23	18	32	50	51	33
Vatican	13	7	6	10	7	7	8	13	9
Venezuela	42	15	3	14	31	11	168	32	40
Viet Nam	124	42	42	44	52	193	47	199	93
Yemen	14	5	33	10	17	9	37	88	27
Zambia	52	58	43	26	12	60	161	187	75
Zimbabwe	84	97	106	86	98	129	264	221	136
Average	61	50	44	39	41	71	81	102	61
